# The Histopathological Findings in Appendectomy Specimens in an Iranian Population

**DOI:** 10.31661/gmj.v12i.2482

**Published:** 2023-10-01

**Authors:** Mahmoud Agholi, Farideh Esfandiari, Hamid Reza Heidarian, Fatemeh Khajeh, Zahra Sharafi, Ehsan Masoudi, Mohammad Rayani

**Affiliations:** ^1^ HIV/AIDS Research Center, Fasa University of Medical Sciences, Fasa, Iran; ^2^ Department of Parasitology and Mycology, Fasa University of Medical Sciences, Fasa, Iran; ^3^ Department of Parasitology and Mycology, School of Medicine, Shiraz University of Medical Sciences, Shiraz, Iran; ^4^ Department of Pathology, Fasa University of Medical Sciences, Fasa, Iran; ^5^ Department of Anatomy, School of Medicine, Shiraz University of Medical Sciences, Shiraz, Iran; ^6^ The Persian Gulf Tropical Medicine Research Center, The Persian Gulf Biomedical Sciences Research Institute, Bushehr University of Medical Sciences, Bushehr, Iran

**Keywords:** Appendicitis, Parasites, Enterobius Vermicularis, Ascaris Lumbricoides, Histopathological Findings

## Abstract

Background: Appendicitis is one of the most common causes of acute abdominal surgeries. The importance of parasitic etiologies in the pathogenesis of appendicitis is not well known in appendectomy specimens on a large scale in southwestern Iran. The current study aimed to retrospectively assess the demographic data and histopathological records of appendicitis in a 28-year period in Fars province, southwestern Iran. Materials and Methods: Histopathological records of 13,013 patients who had undergone surgeries for appendicitis at Dr. Ali Shariati Hospital, affiliated with the Fasa University of Medical Sciences from December 1993 to January 2021 were reviewed and data concerning the patients' demographic data and histopathological records were retrieved from each record. More than 6800 archived microscopic glass slides were also reviewed. Results: From a total of 13,013 histopathological records of surgical excisions of appendicitis that were reviewed over a 28-year period, 8,189 (62.9%) were male and 4,842 (37.1%) were female. Patients' age ranged from 2 to 98 years, with a mean age of 24.68±19.87 years. The most common inflammatory changes were 5,687 (43.7%), 1,228 (9.4%), 670 (5.1%), 522 (4%), and 363 (2.8%) cases of acute appendicitis, suppurative appendicitis, early acute appendicitis, gangrenous appendicitis, and perforated appendicitis respectively. Microscopically, no viral inclusions, fungal elements, and histopathologic findings of bacterial causes were found. Parasitic infections such as helminthiasis were detected in 74 (0.6%) cases aged from 6 to 63. Enterobiasis (Syn. oxyuriasis, pinworm infection) accounted for 73 (98.6%) of the 74 helminthiases, while ascariasis accounted for 1 (1.4%). Out of 74 cases, 29 (39.2%) showed evidence of appendicitis. Conclusion: The results suggest that although parasitic agents are minor causes of appendicitis, these agents should be kept in mind during differential diagnosis. However, whether every parasitic infection leads to appendicitis is controversial.

## Introduction

One of the most prevalent causes of an acute abdomen requiring surgical excision is appendicitis, an inflammation of the vestigial vermiform appendix which is confirmed by histopathologic studies [1]. Many infectious and noninfectious etiologies (e.g., fecaliths, tumors, lymphoid hyperplasia, and so on) are associated with appendicitis [2]. The main cause of acute appendicitis is obstruction of the lumen of the appendix, which affects around 7% of the population. An obstruction caused by parasites in the large intestine and appendix can lead to appendicitis. If the lumen is obstructed by parasites or their ova, appendicitis may develop. The most frequent findings in the majority of investigations on histopathological variations in acute appendicitis due to the parasites were a relatively high frequency of polymorphonuclear leukocytes (PMNs) infiltration and suppurative exudate [3].

Appendicitis can affect anyone of any age, however, it affects men more commonly than women and most cases are seen in patients aged 10 - 40 [1][2]. The function of E. vermicularis in the pathogenesis of appendicitis has been controversial since the first report of the presence of this parasite in the appendix lumen in the late nineteenth century [1][3][4][5][6][7][8][9][10][11][12][13][14][15][16][17][18][19][20][21][22][23][24][25][26][27][28][29][30][31][32][33][34][35][36][37][38][39][40][41][42][43][44][45][46][47][48][49]. Only a few intestinal parasites belonging to both groups, the protozoa and helminths acutely penetrate, or attach to the mucosal lining of the appendicular wall, and can be identified in appendicitis etiology [3]. Giardia lamblia, Entamoeba histolytica, Enterobius vermicularis, Ascaris lumbricoides, Taenia spp., Strongyloides stercoralis, Trichuris trichiura, and Schistosoma spp. are parasites that have been reported can lead to a clinical picture of acute appendicitis. Globally distributed E. vermicularis (Syn. Oxyuris, pinworm), is considered the most frequently parasitic cause of appendicitis [2][3][4]. Although E. vermicularis alone is identified in about 0.2% to 15% of the excised appendices, its actual ability to injure mucosa has widely been discussed [2].

Unfortunately, the importance of parasitic etiologies in the pathogenesis of appendicitis is not well known in appendectomy specimens on a large scale in Iran. This study aimed a retrospective review of tissue sections of appendectomy specimens to determine the prevalence of parasitic agents and their potential involvement in the development of acute appendicitis in southwestern Iran. Thus, the present study was designed and carried out to evaluate the demographic information and histopathological records of appendectomy specimens with a focus on parasitoses-related appendicitis during a 28-year period in Fars region, southwest Iran.

## Materials and Methods

Study Protocol

In this retrospective research, histopathological records of 13,013 appendectomy cases who had undergone surgeries for appendicitis at Dr. Ali Shariati Hospital, affiliated with the Fasa University of Medical Sciences were reviewed and data were retrieved for 28-year, from December 1993 to January 2021 in Fars Province. Therefore, the research team, which included pathologists and parasitologists, searched manually the records. The demographical information, such as age, gender, and histopathological findings of appendectomies were extracted from each patient’s histopathological record. The fundamental basis for the diagnosis of appendicitis was histopathological examinations of the appendectomies. More than 6800 archived microscopic glass slides were also reviewed.

There were no exclusion criteria for this study. Based on whether or not there were parasite structures in the appendix lumen, patients’ pathological records were divided into two groups. Age, gender, and histopathological findings of each case were obtained from the patient’s surgical pathology records.

All the specimens were prepared once longitudinally and twice more transversely. All the available microscopic slides were reviewed and classified by the two parasitologists based on the presence or absence of parasitic agents. The microanatomy, which reveals the parasite structures, was used to diagnose parasites. Cases diagnosed with parasitic agents (e.g., helminths) were then, re-evaluated pathologically for an inflammatory reaction by two expert pathologists. The various stages of acute appendicitis were designated as early acute, acute, suppurative, gangrenous, perforated, and not inflamed vermiform appendix. The diagnosis of acute appendicitis was made when a PMN infiltration was observed in the mucosa or deep layers of the appendix. Eosinophilic appendicitis was also characterized by a diffuse eosinophilic infiltration or by the presence of appendiceal granulomas of epithelial cells, fibroblasts, and many eosinophils having necrotic centers and surrounded by diffuse eosinophilic infiltration. A normal appendix (without inflammation) was defined as one that was conducted to make an acute appendicitis clinical diagnosis, but in which the appendix was found to be normal on histopathologic examination [1]. The number of sections and the gender of helminths were also analyzed.

Statistical Analysis

The information was analyzed using Version 22 of the SPSS software. The Chi-Square and Mann-Whitney tests were utilized for comparison wherever appropriate.

## Results

**Table:** **Table1.** Clinicopathologic Characteristics of the 13,013 Patients Who Had Undergone Appendectomy*

**Patient Characteristics**	**Results**
**Gender**	
Male	8,189 (62.9%)
Female	4,824 (37.1%)
Mean age, y	24.68 ± 11.89
**Histopathologic findings**	
Early acute appendicitis	670 (5.1%)
Acute appendicitis	5,687 (43.7%)
Suppurative appendicitis	1,228 (9.4%)
Gangrenous appendicitis	522 (4%)
Perforated appendicitis	363 (2.8%)
Eosinophilic appendicitis	183 (1.4%)
Granulomatous appendicitis	3 (0.02%)
Chronic appendicitis	41 (0.3%)
Acute on chronic appendicitis	15 (0.1%)
Mucocele	10 (0.1%)
Carcinoid tumor	13 (0.1%)
Mucinous adenoma	13 (0.1%)
Mucinous adenocarcinoma	3 (0.02%)
Lymphoid hyperplasia	200 (1.5%)
Fibrous obliteration	435 (3.3%)
Fecalith only	588 (4.5% )
Diverticula of appendix	7 (0.05%)
Mucosal hyperplasia	3 (0.02%)
Endometriosis	8 (0.1%)
Pseudomxa peritonei	1 (0.01%)
Normal-structured appendix vermiformis**	3,020 (23.4%)
**Total**	13,013 (100%)

^*^ Data are presented as No. (%) and mean ± SD ^**^ As no specific pathologic change

**Figure-1:**
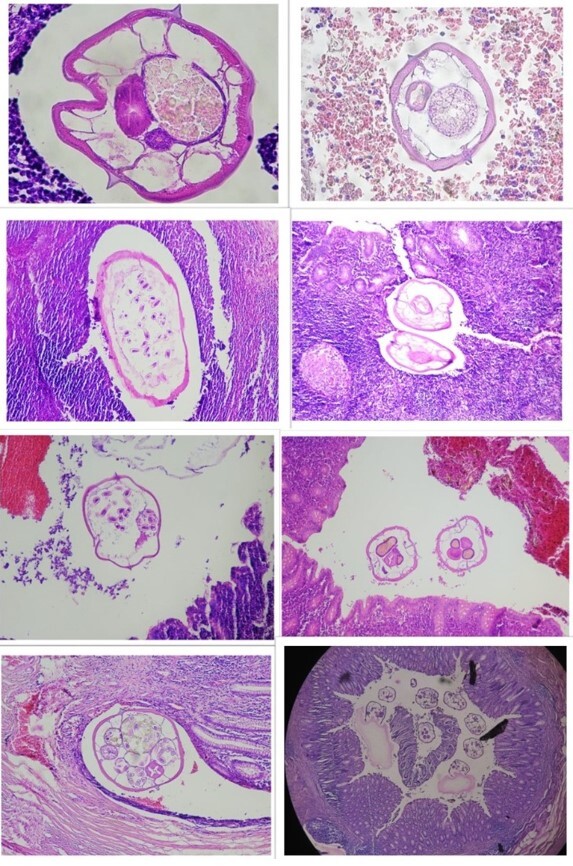
Figure1. Cross-sections of Enterobius vermicularis in the lumens of appendices; H&E stain (×400).

**Table:** **Table2.** Helminthiases* with and without Appendicitis in Relation to Age Groups

**Age group**		**Helminthiases without appendicitis**		**Helminthiases with appendicitis**
	**Male**	**Female**	**Male**	**Female**
≤10	2	3	2	2
11-20	10	17	3	9
21-30	3	4	5	3
31-40	2	2	0	3
41-50	0	1	1	1
51-60	0	0	0	0
61-70	0	1	0	0
**Total**	17	28	11	18

^*^ Helminthiases as 73 enterobiasis and 1 ascariasis

Overall, 8189 (62.9%) patients were male and 4824 (37.1%) were female. Patients’ age ranged from 2 to 98 years and the mean age was 24.68 ± 11.89 years. In histopathologic analysis, 9993 (76.8%) of the specimen showed varying evidence of inflammation or unusual histopathologic findings, giving a 23.4% negative appendectomy rate. The most common inflammatory changes were 5,687 (43.7%), 1,228 (9.4%), 670 (5.1%), 522 (4%), and 363 (2.8%) cases of acute appendicitis, suppurative appendicitis, early acute appendicitis, gangrenous appendicitis, and perforated appendicitis respectively (Table-1).

Microscopically, no viral inclusions, fungal elements, and histopathologic findings of bacterial causes were found microscopically. Parasitic infections were detected in 74 (0.6%). Of 74 parasitoses, 73 (98.6%) were enterobiasis (Figure-1) and 1 (1.4%) was ascariasis. No perforation or gangrene was seen in the specimen containing E. vermicularis. The mean age of patients with parasitic infections was 19.59 ± 10.71 years; 28 (37.8%) were males and 46 (62.2%) were females. Parasites-infected cases were divided into two groups based on whether or not there was inflammation. Group 1 was composed of 45 (60.8%) cases showing only helminth infections without inflammation while the remaining 29 (39.2%) (Group 2) showed both helminth infection and types of inflammation. Parasites-infected patients’ age ranged from 6 to 63 years and acute inflammation was frequently seen in the age group between 11 and 20 years while no case was above 50 years. Microscopic investigation of tissue sections of appendices, including E. vermicularis demonstrated that infection was more frequent in females than in male appendices, with a ratio of 1 male to 1.6 females, indicating that female E. vermicularis was found more frequently than men. There was no correlation between inflammation in the appendix and the gender of E. vermicularis (P˃0.05). Overall, E. vermicularis infection was present in 73 appendices (0.56%); of these, 29 (39.7%) showed a concurrent degree of inflammation, accounting for 0.2% of the study population. Note that in one case, E. vermicularis was associated with granulomatous appendicitis and in another case with eosinophilic appendicitis. Also, early acute appendicitis was seen in the case infected with Ascaris. Totaling, Table-1 shows the clinicopathologic characteristics of patients who had undergone an appendectomy. The distribution of cases infected with parasites by gender, age, and histopathologic findings is summarized in Table-2 and 3.

## Discussion

Enterobiasis or oxyuriasis (Syn. Pinworm infection) is the most common helminthic infection in children of those temperate regions with an estimated 4% to 28% of this age group being affected worldwide [3]. Although it is primarily an infection in young children, rapid maturation of the ova allows the infection to be readily transmitted from child to child and from child to adult, in both family and institutional settings. In different studies, the prevalence of enterobiasis in Iran was reported

**Table:** **Table3.** Histopathologic Findings in Appendectomy Specimens

**Histopathologic findings**	**No. of Male (%)**	**No. of Female (%)**
**Early acute appendicitis**	3 (4.05)	6 (8.1)
**Acute appendicitis**	6 (8.1)	6 (8.1)
**Suppurative appendicitis**	3 (4.05)	2 (2.7)
**Eosinophilic appendicitis**	0 (0)	1 (1.35)
**Granulomatous appendicitis**	0 (0)	1 (1.35)
***Ascaris*** with appendicitis*	0 (0)	1 (1.35)
**Without inflammation**	17 (23)	28 (37.8)
**Subtotal**	29 (39.2)	45 (60.8)
**Total**	74 (100)

among all children, boys, and girls between 1.2%-66.1%, 2.3%-65.5%, and 1.7%-65.5%, respectively [50]. In Iran, the prevalence of pinworm infection in appendicitis is low and there is no statistically significant relation between age or gender and this infection. Iran had a 1% (95 %CI=0.00-0.02) prevalence of E. vermicularis in appendicitis, with the greatest rate being 3% (95%CI=0.02-0.03) and the lowest rate being 0% (95%CI=0.00-0.01) [51]. Females had a substantially higher prevalence of E. vermicularis than males (OR, 0.47; 95%CI, 0.38-0.59). Different behavioral patterns and gender-based differences may be responsible for higher rates of infection in females [2]. The role of this organism in the pathogenesis of appendicitis has been debated since 1899 when the first report of the occurrence of E. vermicularis was published [5]. There is little information available about the inflammatory complications of the vermiform appendix caused by parasite etiologies in Iranian people. Therefore, this retrospective study aimed to determine the prevalence of infectious agents, especially parasites in surgically removed appendices and their possible role in the pathogenesis of appendicitis through histopathological examinations. In this study, cases of parasitic appendicitis were examined and compared to cases in previous studies completed over several years in different regions of the country.

A retrospective study of 1590 removed appendices at Sina, Pars and Imam Khomeini hospitals in Tehran between 1980 and 1990, revealed that 38 (2.39%) tissue specimens had E. vermicularis infections [13]. In another study at Imam Khomeini Hospital in Ahvaz, the capital of Khouzestan Province, Southwestern part of Iran, E. vermicularis was identified in 0.7% of 1,253 surgically removed appendices [19]. Moreover, a histopathological study of removed appendices performed by Mowlavi et al. in Khuzestan province, southwestern Iran, from 2001 to 2003 revealed that all 40 samples were positive for enterobiasis [17]. Also, Fallah and Dehghani reviewed 5,981 appendectomy specimens in the Pathology Department at the two teaching hospitals in Tabriz, northwestern Iran, from 2005 to 2009, and found E. vermicularis in 38 (63.9%) cases [25]. A study by Kazemzadeh et al. revealed that 0.3% of 1,533 surgically removed appendices were infected with pinworm at Al-Zahra Medical Centre in Isfahan, Iran [26]. In addition, a study in Kerman identified E. vermicularis in 0.7% of 5,048 surgically removed appendices from Iranian individuals [24]. Furthermore, in a study conducted in Qom Province in Central Iran between 2005 and 2016, it was observed that there were 31 parasitic appendicitis in 13,477 cases having primary appendicitis [45]. Also, Monajemzadeh et al. reported that 2% of 947 appendectomies were related to infections with E. vermicularis at the Pediatric Center of Excellence, Tehran, Iran between 1988 and 2009 [52]. In another study, Hooshyar et al. reported the case of an 8-year-old girl with acute appendicitis due to hyperinfection with E. vermicularis [1]. Therefore, when determining the differential diagnosis of agents causing appendicitis, it is important to consider the possibility of infection of the appendix with E. vermicularis. Mostly in children, the presence of E. vermicularis in the appendix can cause abdominal pain mimicking features of acute appendicitis or appendiceal colic with no histological evidence of acute inflammation [47].

The presumptive diagnosis of E. vermicularis infection is made clinically based on the history of perianal itching and confirmed by examination of eggs or adults recovered from perianal skin material by the sticky tape technique [50]. In rare cases, this infection has been reported from other uncommon ectopic sites, e.g., the peritoneal cavity, lung, liver, kidney, and fallopian tubes, leading to severe outcomes and even death [2].

In ectopic locations, usually, a degenerating female is found, sometimes only remnants of uteri with eggs, or eggs alone [3]. Pathologic investigation typically reveals a chronic granulomatous inflammation with or without core necrosis that is encircled by neutrophils, eosinophils, and fibroblasts. Charcot-Leyden crystals, giant cells, epithelioid cells, and macrophages may also seen [1]. E. vermicularis can cause signs and symptoms of acute appendicitis that clinically resemble tuberculous lymphadenitis [2].

Since the definitive diagnosis of appendicitis is achieved via histopathologic investigation, appendicitis is one of the most common acute surgical conditions of the abdomen [1][2][3]. Obstruction of the appendix by one of the various causes (e.g., fecaliths, infections such as parasites, tumors, and so on) leads to an increase in luminal and intramural pressure, some with histopathologic findings and others with nonspecific findings that may require a thorough diagnostic assessment [2]. For example, E. vermicularis is found in about 3% of the appendices resected in the United States [1][3]. The parasite is most often found in the appendices of children between the ages of 7 to 11 years [2]. In the present study, depending on whether or not there were parasites in the appendix lumen, the patients were split into two groups: in group 1 (n=74) we observed parasitic infection, whereas in patients of group 2 (n=12,939), no parasitic infestation was present. E. vermicularis was identified in 0.56% (n=73) appendectomy specimens, with previous studies from Iran quoting between 0.2% and 2.9% [13][17][19][24][25][26][45]. This is lower in comparison with the prevalence reported from the conducted studies in some regions of Iran [13][24].

According to several studies, the prevalence of E. vermicularis-infected appendices in other countries was found to be between 0.2% to 15% [5][6][7][8][9][10][11][12][14][15][16][18][20][21][22][23][27][28][29][30][31][32][33][34][35][36][37][38][39][40][41][42][43][44][46][47][48][49]. In recent years, the highest number of pinworm-infected appendectomy specimens (30/200; 15%) was reported from Palestine [41]. However, the worldwide relationship of E. vermicularis infection with acute appendicitis varies widely from 1.74% to 100%, hence making its relationship controversial [2]. Of 73 appendices containing E. vermicularis in this study, 60.3% (44 specimens) belonged to females (Table-2). Despite that, the majority of patients who had undergone appendectomy were male (n=8,189; 62.9%). This finding corresponds to several previous reports from Iran [30][31] and other regions of the world [9][28][42]. It was surprising to note that female pinworms were more commonly seen than males. Although female cases of E. vermicularis infection had a peak age distribution of 11-20 years old, there was no relationship between the gender of appendiceal E. vermicularis and E. vermicularis-infected patients and inflammation (P˃0.05). Furthermore, human infections with E. vermicularis appear to be becoming rarer in Iran. There have been clear decreases in such prevalence over time, probably indicating general improvements in sanitation. Although E. vermicularis infection is the most common helminth parasite in temperate regions [2], this event might explain the lower prevalence of the helminth in the present study.

## Conclusion

Lastly, differential diagnosis of parasitic agents in the etiology of acute appendicitis was done properly in southwestern Iran.This event is the study of appendicitis due to infectious etiologies with a focus on parasitic agents on a large scale in southwestern Iran. Our review of the kinds of the literature shows that the existence of this helminth in the appendiceal lumen can lead to pathologic changes ranging from the normal appendix to acute or suppurative appendicitis, confirmed by our findings.

Data from this study suggest that helminthic agents as the only infectious agents even with the low prevalence can be one of the probable causes of appendicitis etiology in Iran and should be kept in mind during differential diagnosis. We suspect, furthermore, that this number of tissue samples may be the tip of the iceberg, with more cases remaining undetected. However, whether all intestinal parasitic infections lead to inflammation reactions in the appendix response is controversial and requires further investigation. These findings are in line with other studies carried out worldwide.

## Conflict of Interest

The authors state no conflict of interest.
